# Changes in gluten-free diet adherence and growth parameters among children with celiac disease during the COVID-19 pandemic

**DOI:** 10.3389/fped.2026.1923034

**Published:** 2026-09-18

**Authors:** Yağmur Erkol Yılmaz, Ceyda Tuna Kırsaçlıoğlu, Aydan Kansu, Arzu Ensari, Zarife Kuloğlu

**Affiliations:** 1 Department of Pediatrics, School of Medicine, Ankara University, Ankara, Türkiye; 2 Department of Pediatrics, Division of Gastroenterology, Hepatology and Nutrition, School of Medicine, Ankara University, Ankara, Türkiye; 3 Department of Pathology, School of Medicine, Ankara University, Ankara, Türkiye

**Keywords:** celiac disease, COVID-19, gluten-free diet, nutritional status, treatment adherence and compliance

## Abstract

**Objectives:**

Strict exclusion of gluten is the cornerstone of celiac disease (CD) management; nevertheless, maintaining dietary adherence remains challenging. The COVID-19 pandemic may have influenced dietary adherence in children with CD. This study aimed to investigate the association between the COVID-19 pandemic and gluten-free diet (GFD) adherence and anthropometric measurements.

**Methods:**

This retrospective longitudinal cohort study with a cross-sectional survey component included 81 pediatric patients with CD (67.9% female; mean age 14.9 ± 3.4 years) who had been following a GFD for at least one year before the pandemic. Demographic, clinical, laboratory, and anthropometric data from the pre-pandemic, restriction, and post-pandemic periods were obtained from medical records. Dietary adherence was assessed via a structured telephone questionnaire. Participants reporting either complete avoidance of gluten or fewer than six inadvertent gluten exposures annually were categorized as self-reported adherent. Serological adherence was defined by a negative tTG-IgA result.

**Results:**

At the beginning of the pandemic in March 2020, the mean duration of GFD was 3.9 ± 2.4 years. At the time of the survey, self-reported adherence was 67.9% (55/81), whereas serological adherence was 77.1% (54/70). During the pandemic, 49.4% of participants reported no change in dietary adherence, 33.3% reported increased adherence, and 17.3% reported decreased adherence. Serological adherence differed significantly across the three longitudinal assessment periods (Cochran's Q = 31.818, *p* < 0.001). Compared with the pre-pandemic period (51.9%, 42/81), serological adherence was significantly higher during both the restriction period (82.7%, 67/81) and the post-pandemic period (76.5%, 62/81) (McNemar *p* < 0.001 for both comparisons), whereas no significant difference was observed between the restriction and post-pandemic periods (McNemar *p* = 0.227). Serological adherence was associated with home-cooking and selected family and socioeconomic factors. Most participants (87.7%) reported difficulty accessing gluten-free products, mainly due to high cost and limited variety. No significant differences were observed in anthropometric measurements across the study periods.

**Conclusion:**

Serological adherence was significantly higher during both the COVID-19 restriction and post-pandemic periods than during the pre-pandemic period, while no significant changes in anthropometric measurements were observed.

## Introduction

Severe acute respiratory syndrome coronavirus 2 (SARS-CoV-2) first emerged in December 2019, rapidly spread worldwide, and was declared a pandemic by the World Health Organization on March 11, 2020 (1). During the COVID-19 pandemic, public health measures such as isolation, quarantine, curfews, and the closure of schools and workplaces profoundly altered daily life. Children with chronic illnesses may have been particularly affected by these changes.

Celiac disease (CD) is a chronic immune-mediated intestinal disorder that develops following gluten exposure in genetically susceptible individuals. Currently, disease control relies exclusively on strict, lifelong adherence to a gluten-free diet (GFD). Chronic inflammation, malnutrition and concomitant autoimmune diseases have been suggested to increase susceptibility to infections in individuals with CD. Although concerns have been raised regarding susceptibility to COVID-19 among individuals with CD, available studies have generally not demonstrated a consistently increased risk of SARS-CoV-2 infection or severe COVID-19 outcomes, although findings regarding hospitalization risk have varied across cohorts (2–6).

Evidence emerging from different regions has yielded heterogeneous findings regarding dietary adherence during the pandemic period (7–10). Reduced consumption of meals outside the home may have facilitated dietary control, whereas limited access to gluten-free products and economic difficulties may have hindered adherence. In Türkiye, a study among adults with CD reported increased difficulty in accessing gluten-free products; despite this, better adherence was observed among individuals who had followed a GFD for a longer duration (11). However, data regarding the pediatric population are limited, and most available studies rely solely on self-reported adherence without serological confirmation (7–10).

Therefore, this study aimed to evaluate changes in GFD adherence and anthropometric measurements among children with CD across the pre-pandemic, restriction, and post-pandemic periods, using both self-reported and serological measures of adherence, and to identify factors associated with dietary adherence and pandemic-related challenges.

## Methods

This retrospective longitudinal cohort study included a cross-sectional survey component and evaluated changes in GFD adherence and growth parameters among children with CD before, during, and after the COVID-19 pandemic. Longitudinal data were obtained from medical records, while serological adherence at the time of the 2024 survey was evaluated as an additional cross-sectional time point.

## Study population

This study enrolled pediatric patients with CD diagnosed according to the European Society of Pediatric Gastroenterology, Hepatology and Nutrition (ESPGHAN) diagnostic criteria applicable at the time of diagnosis and followed at the Pediatric Gastroenterology Division of Ankara University School of Medicine (12). Eligible patients were younger than 18 years of age during the pandemic, had been on a GFD for at least one year before the COVID-19 pandemic, and had accessible contact information in the hospital medical record system. Demographic data, age at diagnosis, clinical characteristics, anthropometric measurements and type of CD were extracted from patient medical records using a standardized data collection form. CD was classified as classic, non-classic or subclinical according to the Oslo classification (13).

### Data collection and questionnaire

Data collection and analysis were conducted between July 2023 and December 2024. In April 2024, families were contacted by telephone, and a structured questionnaire was administered by a single investigator to minimize inter-observer variability. For participants older than 15 years of age, the questionnaire was answered primarily by the child. For younger participants, or when clarification was needed, the questionnaire was completed jointly by the parent and the child. The questionnaire collected information on place of residence, parental education and health status, socioeconomic level, presenting symptoms, comorbidities, duration of GFD, current dietary adherence, and pandemic-related experiences. For the longitudinal analyses, the duration of the gluten-free diet was calculated from the documented date of diet initiation to March 2020, corresponding to the beginning of the COVID-19 pandemic. Participant age was calculated at the time of the April 2024 survey. Pandemic-specific questions addressed changes in dietary adherence, access to gluten-free products, home-cooking habits, COVID-19 infection history, vaccination status, outpatient clinic attendance, and engagement with activities organized by the Celiac Life Association. Pandemic-related changes in self-reported GFD adherence were assessed using a three-category response (increased, decreased, or unchanged). Participants who reported an increase or decrease in GFD adherence were subsequently asked to identify perceived reasons for this change from predefined response options, with more than one response permitted. Examples of pre-determined positive factors included, better eating habits at home, better ability to follow the diet when working/staying at home, healthier snack habits, an increase in the amount of fruits and vegetables consumed, spending more time with the child and/or mother, having more involvement in preparing meals for family members, reducing the number of times family members ate outside the house. Predefined negative factors included poor eating habits at home, difficulty adhering to the diet while working/staying at home, increased consumption of snacks/junk foods, difficulty obtaining and higher costs associated with gluten-free products; economic difficulties due to the pandemic; increased feelings of anxiety/distress related to the pandemic; accidental exposure to gluten through shared meals at home. Changes in home preparation of gluten-free meals were separately categorized as increased, decreased, or unchanged. Access to gluten-free products was assessed using predefined categories ranging from no difficulty to inability to obtain products, and participants reporting difficulties could select multiple reasons, including product unavailability, high cost, limited variety, and avoidance of crowded places. These questionnaire-derived variables were analyzed as categorical variables.

June 2021 was selected because nationwide COVID-19 restrictions in Türkiye had largely been lifted by that time, whereas June 2022 represented a later follow-up period after normalization of daily life and healthcare services. Family structure was categorized according to household composition. A nuclear family was defined as a household consisting of the mother, father, and no more than two children. Households with three or more children and/or additional family members living in the household were categorized as extended families.

### Assessment of dietary adherence

Self-reported adherence was assessed using participants' responses to three adherence categories—excellent, good, or poor—and their reported frequency of consumption of gluten-containing foods. Patients reporting no gluten intake or fewer than six gluten exposures per year were considered self-reported adherent. This threshold was based on clinical judgment and previous observational practice; however, it does not represent a validated adherence instrument such as the Biagi score. Serological adherence was defined as a negative tTG-IgA result at the relevant assessment time point. All tTG-IgA measurements were performed in the same clinical laboratory at Ankara University Children's Hospital using enzyme-linked immunosorbent assay (ELISA). During the study period, different commercially available ELISA assay systems were used as part of routine laboratory practice. These included the ORGENTEC Diagnostika (Mainz, Germany) assay performed on the ETI-MAX 3000 platform (positivity cut-off >10 U/mL) and the EUROIMMUN (Lübeck, Germany) assay performed on the Analyzer-2 platform (positivity cut-off >20 RU/mL). Serological adherence was classified according to the laboratory-defined, assay-specific positivity threshold applicable to each measurement. None of the participants had selective IgA deficiency.

### Anthropometric assessment

Anthropometric data were obtained from patient medical records at three predefined time points: the last outpatient visit before March 2020 (*pre-pandemic period*), the first visit after June 2021 (*pandemic restriction period)*, and the first visit after June 2022 (*post-pandemic period*). Length/height, weight, and body mass index (BMI) were recorded and z-scores calculated using the CEDD Çözüm online growth assessment system, which implements the Neyzi growth references for Turkish children (14). Short stature was defined as height-for-age (HFA) z score < −2, underweight as weight-for-age (WFA) z score < −2, thinness as BMI z score < −2, and obesity as BMI z score > + 2. Malnutrition was defined as HFA and/or BMI z score < −2 (15).

### Statistical analysis

All statistical analyses were performed using SPSS (version 30.0; IBM Corp., Chicago, IL, USA). Descriptive statistics were presented as mean ± standard deviation or median (minimum–maximum), depending on the distribution of the data, and as frequency (percentage) for categorical variables. The normality of continuous variables was assessed using the Shapiro–Wilk test. For the analysis of continuous variables across repeated measurements, repeated measures ANOVA was used for normally distributed data, while the Friedman test was applied for non-normally distributed variables. The Kruskal–Wallis test was used for comparisons between independent groups where appropriate. *Post-hoc* pairwise comparisons were performed where appropriate. Categorical variables were compared using the chi-square test or Fisher's exact test, as appropriate. Age, sex, and GFD duration were examined as potential factors associated with adherence in univariable and subgroup analyses. Given the relatively small sample size, multivariable regression analysis was not performed to avoid model overfitting. Changes in serological adherence across the three longitudinal assessment periods were evaluated using Cochran's *Q* test. When the overall test was statistically significant, pairwise comparisons were performed using McNemar's test with Bonferroni correction for multiple comparisons. Exact McNemar probabilities were used when appropriate. For the three pairwise comparisons, the Bonferroni-adjusted significance threshold was set at *p* < 0.0167. For all other analyses, a two-sided *p* value < 0.05 was considered statistically significant.

### Ethics statement

The study protocol was approved by the Ankara University Faculty of Medicine Human Research Ethics Committee (Approval No: I06-371-23) and was conducted in accordance with the Declaration of Helsinki and relevant institutional guidelines. Retrospective clinical data were obtained from hospital records. For the questionnaire component conducted in April 2024, participants aged 18 years or older provided written informed consent themselves. For participants younger than 18 years, written informed consent was obtained from a parent or legal guardian, and age-appropriate assent was obtained from the participant.

## Results

### Participant characteristics

Of 105 eligible patients identified, 81 were included in the final analysis after exclusions due to incomplete data or inability to contact families (Figure 1). The demographic, clinical, and socioeconomic characteristics of the cohort are presented in Table 1.

**Figure 1:**
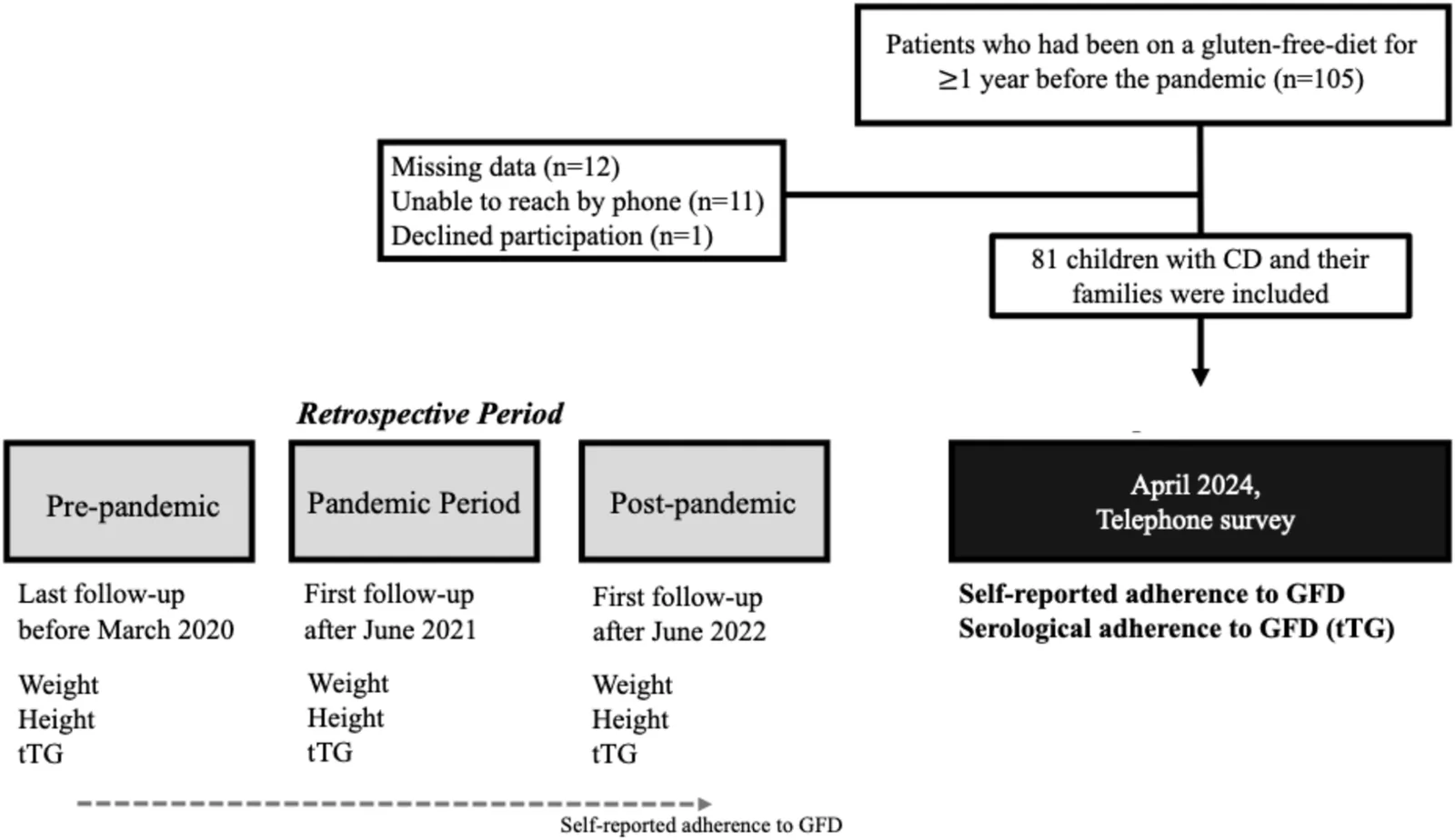
Study flow diagram illustrating patient selection and the number of participants included. CD, celiac disease; GFD, gluten-free diet; TTG, tissue transglutaminase.

**Table 1:** Demographic, clinical, and socioeconomic characteristics.

Characteristic	Total (*n* = 81)
Age at the time of the survey, years^a^	14.9 ± 3.4 (6.3 to 20.1)
Sex, female *n* (%)	55 (67.9)
Age at symptom onset, months^a^	75.9 ± 46.5 (6 to 180)
Age at diagnosis, months^a^	84.5 ± 44.0 (12 to 180)
Disease type, *n* (%)	
Classical	24 (29.6)
Non-classical	43 (53)
Subclinical	14 (17.3)
Comorbidities, *n* (%)	30 (37)
Place of residence, *n* (%)	
Same city as the follow-up center	70 (86.4)
Different city than the follow-up center	11 (13.6)
Education level, *n* (%)	
Mother	
Illiterate	3 (3.7)
Primary school	33 (40.7)
High school	31 (38.3)
University	14 (17.3)
Father	
Illiterate	1 (1.2)
Primary school	28 (34.6)
High school	29 (35.8)
University	23 (28.4)
Family structure, n (%)	
Nuclear family	54 (66.7)
Extended family	27 (33.3)
Income level, n (%)	
Below minimum wage	1 (1.2)
One to two minimum wages	24 (29.6)
Above two minimum wages	56 (69.1)
Family history of celiac disease, n (%)	8 (9.8)
Another household member on a gluten-free diet, n (%)	22 (27.2)

a Data are presented as mean ± SD and range in parentheses*.*

### Gluten-free diet adherence at the time of the survey

At the time of the survey, self-reported adherence was observed in 55 of the 81 participants (67.9%). Among the 54 participants from nuclear families, 43 (79.6%) were self-reported adherent, compared with 12 of the 27 participants (44.4%) from extended families (*p* = 0.002). Serological data at the time of the survey were available for 70 participants. Among the 44 participants from nuclear families with available serological data, 38 (86.4%) were serologically adherent, compared with 16 of the 26 participants (61.5%) from extended families (Fisher's exact test, *p* = 0.037). Overall, 54 of the 70 participants (77.1%) were serologically adherent. Factors affecting GFD adherence at the time of the survey are detailed in Supplementary Table 1. Most participants (87.7%) reported difficulties accessing gluten-free products, primarily because of high product costs (59.2%) and limited product variety (50.7%). Participants could report more than one reason for difficulty accessing gluten-free products.

### COVID-19-related findings and changes in adherence

During the pandemic, 28.4% of participants had polymerase chain reaction (PCR) confirmed COVID-19. All but one infected participant (95.7%) had mild disease. The vaccination rate was 13.6%, with 10 patients receiving BioNTech and one receiving SinoVac. During the pandemic, more than half of patients (55.6%) did not attend routine outpatient clinic visits. A perceived increased risk of COVID-19 infection related to CD was reported by 87.7% of the participants. Regarding dietary adherence***,*** 49.4% of participants reported no change, 33.3% reported an increase, and 17.3% reported a decrease.

Serological adherence differed significantly across the three longitudinal assessment periods (Cochran's Q = 31.818, df = 2, *p* < 0.001). Adherence increased from 51.9% (42/81) in the pre-pandemic period to 82.7% (67/81) during the restriction period and was 76.5% (62/81) in the post-pandemic period. In Bonferroni-adjusted pairwise comparisons, serological adherence was significantly higher during both the restriction and post-pandemic periods than during the pre-pandemic period (McNemar *p* < 0.001 for both comparisons). No significant difference was observed between the restriction and post-pandemic periods (exact McNemar *p* = 0.227). At the time of the survey, serological adherence was 77.1% (54/70), with serological data unavailable for 11 participants (Figure 2). All retrospective serological measurements were available for all 81 participants, whereas survey-period serological data were available for 70 participants.

**Figure 2:**
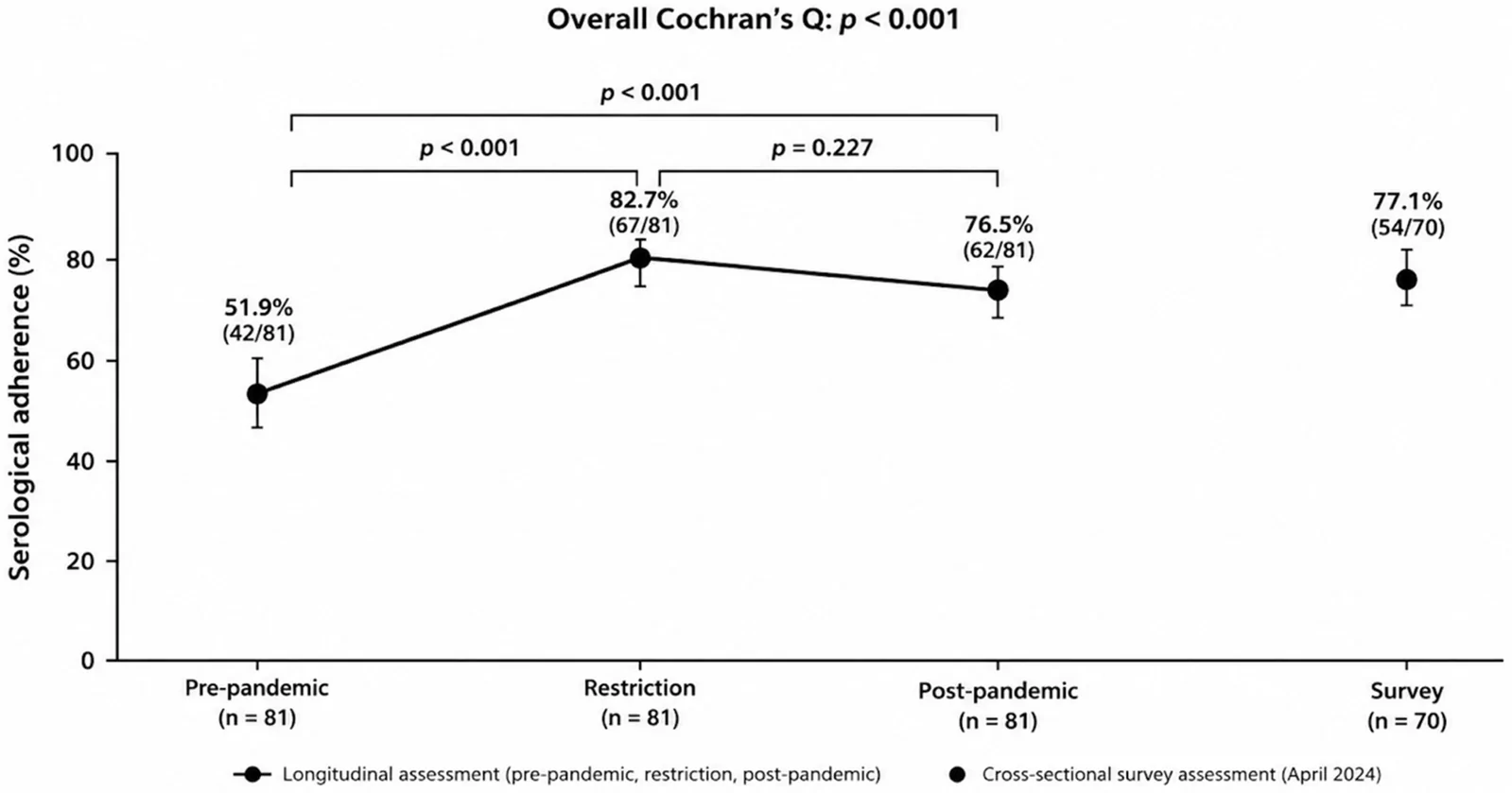
Serological adherence rates across the pre-pandemic, restriction, post-pandemic, and survey assessments.

*Serological adherence was defined as a negative tTG-IgA result according to the assay-specific laboratory reference threshold applicable at each assessment. Survey-period serology was available for 70 participants and was not included in the longitudinal inferential analysis.*

During the pandemic, 14.8% of participants reported an increase in CD-related symptoms. Among these participants, 66.7% were unable to attend regular follow-up visits and 50.0% reported decreased GFD adherence. Notably, 92.6% of participants who reported increased adherence reported no symptom exacerbation, whereas 42.9% of those with decreased adherence experienced worsening symptoms (*p* = 0.007).

### Factors associated with adherence to the gluten-free diet during the COVID-19 pandemic

Among the 27 participants who reported increased self-reported adherence during the pandemic, 15 (55.6%) belonged to nuclear families, whereas 12 (44.4%) belonged to extended families. These proportions refer to the distribution of participants reporting increased adherence rather than adherence rates within each family structure. Serological adherence was higher among children from nuclear families and was associated with home-cooking, family-related factors, and maternal education level. Serological adherence differed significantly according to maternal education level during both the restriction period (*p* = 0.043) and the post-pandemic period (*p* = 0.002). However, the small number of participants in some educational subgroups, particularly the illiterate group, limits interpretation of these findings. Detailed results are presented in Supplementary Table 2.

Factors perceived to facilitate adherence included better control of food choices at home (*p* < 0.001), staying at home (*p* < 0.001), healthier eating habits (*p* < 0.001), reduced meals outside the home (*p* < 0.001), and spending more time with their mothers (*p* < 0.001). Perceived barriers included staying at home (*p* < 0.001), economic challenges (*p* < 0.001), limited product availability and rising costs (*p* < 0.001), and increased consumption of snacks and junk food (*p* < 0.001). Less frequent barriers included pandemic-related stress (*p* = 0.004), unhealthy eating habits (*p* = 0.028), and accidental gluten exposure from shared meals (*p* = 0.028). Overall, 35.8% of families reported increased home-cooking, which was strongly associated with both self-reported and serological adherence (*p* < 0.001). In particular, home meal preparation was positively associated with serological adherence during the restriction and post-pandemic periods.

Difficulties accessing gluten-free products were reported by 87.7% of participants (*n* = 71), primarily due to high product cost (40.8%), limited product variety (28.1%), unavailability of products in markets (16.9%), and avoidance of crowded places (15.5%). Compared with the pandemic period, a significant increase was observed in the number of patients reporting difficulties due to both high product costs (40.8% vs. 59.2%; *p* = 0.001) and limited product variety (28.1% vs. 50.7%; *p* = 0.002) at the time of the survey.

### Anthropometric findings

No significant differences were observed in height, weight, and BMI z-scores across the three time periods (Table 2). The prevalence of malnutrition was 17.3% before the pandemic, remained unchanged during the restriction period, and increased to 19.8% by the end of the pandemic; however, this increase did not reach statistical significance.

**Table 2:** Anthropometric measurements and nutritional status of children with celiac disease during the COVID-19 pandemic.

Anthropometric parameter	Pre-pandemic period	Restriction period	Post-pandemic period	*p*
Weight-for-age z score^a^	−0.58 ± 1.23	−0.59 ± 1.37	−0.57 ± 1.43	0.895
< −2 *n*, (%)	9/81, (11.1)	11/81, (13.5)	13/81, (16.0)	
−2 to +2 *n*, (%)	70/81, (86.4)	68/81, (84.0)	66/81, (81.5)	
> + 2 *n*, (%)	2/81, (2.5)	2/81, (2.5)	2/81, (2.5)	
Height-for-age z score^a^	−0.22 ± 1.17	−0.26 ± 1.13	−0.16 ± 1.10	0.132
< −2 *n*, (%)	5/81, (6.2)	4/81, (4.9)	5/81, (6.2)	
−2 to +2 *n*, (%)	75/81, (92.6)	77/81, (95.1)	76/81, (93.8)	
> + 2 *n*, (%)	1/81, (1.2)	0/81, (0)	0/81, (0)	
BMI z score^a^	−0.59 ± 1.23	−0.58 ± 1.36	−0.62 ± 1.51	0.862
< −2 *n*, (%)	10/81, (12.4)	12/81, (14.8)	13/81, (16.0)	
−2 to +2 *n*, (%)	70/81, (86.4)	68/81, (84.0)	68/81, (84.0)	
> + 2 *n*, (%)	1/81, (1.2)	1/81, (1.2)	0/81, (0)	
Nutritional Status, *n* (%)				
Normal	66/81, (81.5)	66/81, (81.5)	65/81, (80.2)	
Malnutrition	14/81, (17.3)	14/81, (17.3)	16/81, (19.8)	
Obesity	1/81, (1.2)	1/81, (1.2)	0/81, (0)	

BMI, body-mass index.

a Data are presented as mean ± SD.

## Discussion

In this study, changes in GFD adherence and nutritional status across the COVID-19 pandemic periods were evaluated in children with CD. Unlike previous studies of GFD adherence during the pandemic that relied primarily on self-reported adherence data (7–10), our study combined self-reported and longitudinal serological measures. Serological adherence differed significantly across the three longitudinal assessment periods, with significantly higher rates during both the restriction and post-pandemic periods than during the pre-pandemic period. No significant difference was observed between the restriction and post-pandemic periods, whereas no significant changes were observed in height, weight, or BMI z-scores. In addition, several household, behavioral, and socioeconomic factors were associated with adherence. Notably, increased home meal preparation during the pandemic was significantly associated with both higher rates of self-reported adherence and serological adherence. An important contribution of this study is the inclusion of longitudinal serological data alongside self-reported adherence measures.

Published studies addressing dietary adherence to a GFD among patients with CD during the COVID-19 pandemic have yielded inconsistent conclusions (7–10). In our study, serological adherence increased from 51.9% in the pre-pandemic period to 82.7% during the restriction period and was 76.5% in the post-pandemic period. Pairwise analyses demonstrated significantly higher adherence during both the restriction and post-pandemic periods than during the pre-pandemic period, whereas no significant difference was observed between the restriction and post-pandemic periods. At the time of the survey, self-reported adherence was 67.9%, whereas serological adherence was 77.1%. Interestingly, serological adherence exceeded self-reported adherence, which differs from the pattern commonly expected with subjective adherence assessment. One possible explanation is that families may have reported occasional or suspected gluten exposures despite negative serological findings. In addition, serological markers reflect longer-term dietary exposure, whereas self-reported measures may be more sensitive to recent behaviors and perceived dietary lapses.

An additional consideration is the natural decline of tTG-IgA concentrations following the initiation of a gluten-free diet. Because participants had been following a gluten-free diet for varying durations before the pandemic, gradual serological normalization over time may have contributed to the higher serological adherence rates observed during the restriction and post-pandemic periods. Therefore, the observed differences in serological adherence rates should be interpreted cautiously and cannot be attributed solely to the effects of the COVID-19 pandemic. Although this suggests that GFD duration alone may not fully account for the observed differences, natural serological normalization remains an important potential contributor.

The second principal outcome of our study was the evaluation of anthropometric changes across the pandemic periods. Lifestyle changes during the COVID-19 pandemic have been widely reported to adversely affect the nutritional status of children. Reduced physical activity, increased screen time, and higher consumption of calorie-dense foods have been strongly linked to weight gain (16–20), especially among children who were overweight or obese (17, 18). In contrast, no significant changes in height, weight, or BMI z-scores were observed across the pre-pandemic, restriction, and post-pandemic periods in our cohort. A small, statistically non-significant increase in the prevalence of malnutrition was noted. Changes in linear growth may require a longer duration of sustained nutritional change than was captured during the restriction period, which may partly explain the absence of detectable changes in height z-scores. Overall, our findings indicate that anthropometric parameters remained relatively stable across the study periods. However, interpretation is limited by the retrospective collection of anthropometric measurements and the relatively small sample size.

Several household and socioeconomic factors were associated with adherence in our cohort. Family structure was associated with dietary adherence, with higher self-reported and serological adherence rates observed among participants living in nuclear families. This association may reflect differences in household food preparation, supervision, or the complexity of maintaining a strict GFD in larger households. However, detailed household dynamics were not assessed, and the mechanisms underlying this association cannot be determined from our data. Maternal education level was also associated with serological adherence during the restriction and post-pandemic periods. However, the pattern was not linear across educational categories, and some subgroups contained very few participants; these findings should therefore be interpreted cautiously. Although maternal education has previously been associated with dietary adherence (21), our study did not collect information on maternal employment, working conditions, or time spent at home, which may also have influenced family support and meal preparation during the pandemic. A recent study by Saed et al. further demonstrated that adherence may be influenced not only by demographic and economic circumstances but also by patients' attitudes toward the GFD (22). Although their study involved adults, their findings highlight the potential value of assessing the attitudes and beliefs of both children and caregivers using validated instruments in future pediatric studies.

Participants also reported several perceived facilitators and barriers to GFD adherence during the pandemic. Facilitators included staying at home, greater control over food choices, healthier eating behaviors, reduced consumption of meals outside the home, and increased time spent with family members. Conversely, prolonged time at home was perceived as a barrier by some participants, while economic difficulties, psychosocial stress, unhealthy eating behaviors, accidental gluten exposure, and difficulties accessing gluten-free products were also reported as barriers. These findings suggest that pandemic-related circumstances may have had heterogeneous effects on dietary behavior across families. Importantly, difficulties in accessing gluten-free products remained common at the time of the survey, with 87.7% of participants reporting access problems, particularly high cost and limited product variety. These findings highlight the potential challenge of sustaining strict GFD adherence when families face persistent economic and food-access barriers. Strategies aimed at improving availability and affordability of gluten-free products may therefore be important for long-term dietary management.

Healthcare disruption represented an additional challenge during the pandemic, with more than half of the participants missing routine follow-up visits. This finding underscores the potential role of telemedicine, digital monitoring, and remote dietary counseling in supporting continuity of care for children with CD when face-to-face follow-up is disrupted (23). Evidence suggests that telephone-based follow-up and telemedical nutritional interventions may support dietary adherence in selected patient populations (24, 25).

COVID-19 infection and vaccination were secondary outcomes in this study. Among participants with documented SARS-CoV-2 infection, most experienced a mild disease course, and none required intensive care or died due to COVID-19. These findings are broadly consistent with pediatric reports suggesting favorable COVID-19 outcomes among children with CD (26). The vaccination rate in our cohort was 13.6%. Interpretation of this proportion requires consideration of age-specific vaccine eligibility and vaccine availability during the study period. In a multicenter pediatric study from Türkiye conducted in February 2022, vaccination coverage was 12.5% among all children with COVID-19 but 38.7% among those older than 12 years who were eligible for vaccination (27). In comparison, a later Italian pediatric CD cohort reported that 44.8% of patients had received at least one SARS-CoV-2 vaccine dose, with substantially higher coverage among adolescents (28). Differences in study period, age distribution, eligibility criteria, and vaccination policies limit direct comparison between these cohorts.

The strengths of this study include the combined assessment of dietary adherence using both self-report and serological measures, evaluation of multiple influencing factors, and the administration of the questionnaire by a single researcher, thereby reducing inter-observer variability. However, several limitations of this study should be acknowledged. First, the single-center design and relatively small sample size may limit the generalizability of the findings to broader populations. Second, the reliance on retrospective medical records and patient recall for self-reported adherence introduces potential recall bias and measurement variability across time points. In addition, the inability to contact all eligible patients raises the possibility of selection bias. The use of different data sources, including medical records for earlier periods and telephone-based questionnaires for the survey phase, may have introduced measurement heterogeneity. Because of the retrospective design, temporal changes in routine laboratory assay systems could not be controlled; however, serological adherence was classified according to the laboratory-defined, assay-specific positivity threshold applicable at each assessment. Furthermore, the definition of self-reported adherence was not based on a validated scoring system, which may affect the comparability of the findings with other studies. Due to the limited sample size, multivariable analyses were not performed, restricting the ability to adjust for potential confounding factors. Survey-period serological data were unavailable for 11 participants, which may have introduced additional selection bias at the final assessment. Moreover, because tTG-IgA concentrations generally decline over time after the initiation of a GFD, part of the observed differences in serological adherence rates may reflect natural normalization rather than pandemic-related behavioral changes. In addition, the questionnaire-derived facilitators and barriers were based on predefined, non-validated items and should therefore be interpreted as exploratory perceptions rather than validated determinants of adherence. These limitations restrict causal interpretation and should be considered when generalizing the findings.

## Conclusion

Serological adherence was significantly higher during both the COVID-19 restriction and post-pandemic periods than during the pre-pandemic period, with no significant difference between the restriction and post-pandemic periods. No significant changes in anthropometric measurements were observed across the study periods.

## Data Availability

The raw data supporting the conclusions of this article will be made available by the authors, without undue reservation.
