# Supplementary material for: Changes in gluten-free diet adherence and growth parameters among children with celiac disease during the COVID-19 pandemic
Source: Front Pediatr. 2026 Sep 18;14:1923034. doi: 10.3389/fped.2026.1923034 (PMC13631045; doi:10.3389/fped.2026.1923034)
Supplement: Supplementary file 1 [file Table1.docx]

**Table S1. Factors affecting gluten free diet adherence at the time of the survey**

|  |  | **Verbally**  **n, (%)** | | **Serological**  **n, (%)** | | | | | |
| --- | --- | --- | --- | --- | --- | --- | --- | --- | --- |
| **Age** |  | **Adherent** | **Non-adherent** | **Adherent** | | **Non-adherent** | | | **Missing data** |
|  | ***<10 years (n=7)*** | 4, (57.1) | 3, (42.9) | 6, (85.7) | | 1, (14.3) | | | 0, (0) |
|  | ***>10 years (n=74)*** | 51, (68.9) | 23, (31.1) | 48, (64.9) | | 15, (20.2) | | | 11, (14.9) |
|  | ***p*** | n.s | | n.s | | | | | |
| **Sex** | ***Female (n=55)*** | 36, (65.5) | 19, (34.5) | 35, (63.6) | | 13, (23.6) | | | 7, (12.7) |
|  | ***Male (n=26)*** | 19, (73.1) | 7, (26.9) | 19, (73.1) | | 3, (11.5) | | | 4, (15.4) |
|  | ***p*** | n.s | | n.s | | | | | |
| **Disease type** | ***Classical (n=24)*** | 19, (79.2) | 5, (20.8) | 18, (75) | | 4, (16.7) | | | 2, (8.3) |
|  | ***Non-classical (n=43)*** | 25, (58.1) | 18, (41.9) | 26, (60.5) | | 12,( 27.9) | | | 5, (11.6) |
|  | ***Subclinical (n=14)*** | 11, (78.6) | 3, (21.4) | 10, (71.4) | | 0, (0) | | | 4, (28.6) |
|  | ***p*** | n.s | | n.s | | | | | |
| **Household income** | ***Below minimum. wage (n=1)*** | 0, (0) | 1, (100) | 1, (100) | | | 0, (0) | 0, (0) | |
|  | ***One to two min. wages (n=24)*** | 16, (66.7) | 8, (33.3) | 15, (62.5) | | | 9, (37.5) | 0, (0) | |
|  | ***Above two min. wages (n=56)*** | 39, (69.6) | 17, (30.4) | 38, (67.9) | | | 7, (12.5) | 11, (19.6) | |
|  | ***p*** | n.s | | n.s | | | | | |
| **Family structure** | ***Extended (n=27)*** | 12, (44.4) | 15, (55.6) | 16, (59.3) | | | 10, (37) | 1, (3.7) | |
|  | ***Nuclear (n=54)*** | 43, (79.6) | 11, (20.4) | 38, (70.4) | | | 6, (11.1) | 10, (18.5) | |
|  | ***p*** | **0.002** | | **0.037** | | | | | |
| **Family history of CD** | ***Present (n=14)*** | 8, (57.1) | 6, (42.9) | 11, (78.6) | | | 1, (7.1) | 2, (14.3) | |
|  | ***Absent***  ***(n=67)*** | 47, (70.1) | 20, (29.9) | 43, (64.2) | | | 15, (22.4) | 9, (13.4) | |
|  | ***p*** | n.s | | n.s | | | | | |
| **A household member following a GFD** | **Present *(n=22)*** | 15, (68.2) | 7, (31.8) | 18, (81.8) | 1, (4.5) | | | 3, (13.6) | |
|  | **Absent**  ***(n=59)*** | 40, (67.8) | 19, (32.2) | 36, (61) | 15, (25.4) | | | 8, (13.6) | |
|  | ***p*** | n.s | | n.s | | | | | |

*(n.s, non-significant), CD, celiac disease; GFD, gluten-free diet*

**Table S2. Factors affecting serological adherence to the gluten-free diet during the pandemic**

|  |  | **Serological adherence to gluten-free diet**  **n, (%)** | | | | | |
| --- | --- | --- | --- | --- | --- | --- | --- |
|  |  | **Pre-pandemic period** | | **Restriction period** | | **Post-pandemic period** | |
|  |  | **Adherent** | **Non-adherent** | **Adherent** | **Non-adherent** | **Adherent** | **Non-adherent** |
| **Age** | ***<10 years, (n=31)*** | 18, (58.1) | 13, (41.9) | 27, (87.1) | 4, (12.9) | 25, (80.6) | 6, (19.4) |
|  | ***>10 years, (n=50)*** | 24, (48) | 26, (52) | 40, (80) | 10, (20) | 37, (74) | 13, (26) |
|  | ***p*** | n.s | | n.s | | n.s | |
| **Sex** | ***Female, (n=55)*** | 27, (49.1) | 28, (50.9) | 44, (80) | 11, (20) | 41, (74.5) | 14, (25.5) |
|  | ***Male, (n=26)*** | 15, (57.7) | 11, (42.3) | 23, (88.5) | 3, (11.5) | 21, (80.8) | 5, (19.2) |
|  | ***P*** | n.s | | n.s | | n.s | |
| **Disease type** | ***Classical, (n=24)*** | 13, (54.2) | 11, (45.8) | 22, (91.7) | 2, (8.3) | 18, (75) | 6, (25) |
|  | ***Non-classical, (n=43)*** | 20, (46.5) | 23, (53.5) | 33, (76.7) | 10, (23.3) | 32, (74.4) | 11, (25.6) |
|  | ***Subclinical, (n=14)*** | 9, (64.3) | 5, (35.7) | 12, (85.7) | 2, (14.3) | 12, (85.7) | 2, (14.3) |
|  | ***p*** | n.s | | n.s | | n.s | |
| **GFD duration** | ***< 3 years, (n=36)*** | 18, (50) | 18, (50) | 29, (80.6) | 7, (19.4) | 26, (72.2) | 10, (27.8) |
|  | ***≥ 3 years, (n=45)*** | 24, (53.3) | 21, (46.7) | 38, (84.4) | 7, (15.6) | 36, (80) | 9, (20) |
|  | ***p*** | n.s | | n.s | | n.s | |
| **Home Cooking** | ***Decreased, (n=2)*** | 0,(0) | 2, (100) | 0, (0) | 2, (100) | 0, (0) | 2, (100) |
|  | ***No change, (n=50)*** | 27,(54) | 23, (46) | 46, (92) | 4, (8) | 44, (88) | 6, (12) |
|  | ***Increased, (n=29)*** | 15,(51.7) | 14, (48.3) | 21, (72.4) | 8, (27.6) | 18, (62) | 11, (38) |
|  | ***p*** | n.s | | ***<0.001*** | | ***<0.001*** | |
| **Comorbidity** | ***Present, (n=30)*** | 14, (46.7) | 16, (53.3) | 22, (73.3) | 8, (26.7) | 21, (70) | 9, (30) |
|  | ***Absent, (n=51)*** | 28, (54.9) | 23, (45.1) | 45, (88.2) | 6, (11.8) | 41, (80.4) | 10, (19.6) |
|  | ***p*** | n.s | | n.s | | n.s | |
| **Family structure** | ***Extended (n=27)*** | 10, (37) | 17, (63) | 21 ,(77.8) | 6, (22.2) | 18,(66.7) | 9, (33.3) |
|  | ***Nuclear (n=54)*** | 32, (59.3) | 22, (40.7) | 46, (85.2) | 8, (14.8) | 44,(81.5) | 10, (18.5) |
|  | ***p*** | n.s | | n.s | | n.s | |
| **CD in family** | ***Present, (n=14)*** | 9, (64.3) | 5, (35.7) | 14, (100) | 0, (0) | 14, (100) | 0, (0) |
|  | ***Absent, (n=67)*** | 33, (49.3) | 34, (50.7) | 53, (79.1) | 14, (20.9) | 48, (71.6) | 19, (28.4) |
|  | ***p*** | n.s | | n.s | | ***0.032*** | |
| **A household member following a GFD** | ***Present, (n=22)*** | 12, (54.5) | 10, (45.5) | 21, (95.5) | 1, (4.5) | 22 (100) | 0, (0) |
|  | ***Absent, (n=59)*** | 30, (50.8) | 29, (49.2) | 46, (78) | 13, (22) | 40, (67.8) | 19, (32.2) |
|  | ***p*** | n.s | | n.s | | ***0.002*** | |
| **Maternal education level** | ***Illiterate, (n=3)*** | 1, (33.3) | 2, (66.7) | 1, (33.3) | 2, (66.7) | 0, (0) | 3, (100) |
|  | ***Primary school, (n=33)*** | 17, (51.5) | 16, (48.5) | 27, (81.8) | 6, (18.2) | 23, (69.7) | 10, (30.3) |
|  | ***High school, (n=31)*** | 16, (51.6) | 15, (48.4) | 25, (80.6) | 6, (19.4) | 25, (80.6) | 6, (19.4) |
|  | ***University (n=14)*** | 8, (57.1) | 6, (42.9) | 14, (100) | 0, (0) | 14, (100) | 0, (0) |
|  | ***p*** | n.s | | ***0.043*** | | ***0.002*** | |
| **Paternal education level** | ***Illiterate, (n=1)*** | 0, (0) | 1, (100) | 0, (0) | 1, (100) | 0, (0) | 1, (100) |
|  | ***Primary school, (n=28)*** | 12, (42.9) | 16, (57.1) | 21, (75) | 7, (25) | 19, (67.9) | 9, (32.1) |
|  | ***High school, (n=29)*** | 16, (55.2) | 13, (44.8) | 25, (86.2) | 4, (13.8) | 22, (75.9) | 7, (24.1) |
|  | ***University (n=23)*** | 14, (60.9) | 9, (39.1) | 21, (91.3) | 2, (8.7) | 21, (91.3) | 2, (8.7) |
|  | ***p*** | n.s | | n.s | | n.s | |
| **The activities of the Celiac Life Association** | ***Follower, (n=6)*** | 2, (33.3) | 4, (66.7) | 5, (83.3) | 1, (16.7) | 4, (66.7) | 2, (33.3) |
|  | ***Non-follower (n=75)*** | 40, (53.3) | 35, (46.7) | 62, (82.7) | 13, (17.3) | 58, (77.3) | 17, (22.7) |
|  | ***p*** | n.s | | n.s | | n.s | |
| **Pandemic-related anxiety** | ***No anxiety, (n=10)*** | 6, (60) | 4, (40) | 7, (70) | 3, (30) | 7, (70) | 3, (30) |
|  | ***Mild, (n=38)*** | 18, (47.4) | 20, (52.6) | 33, (86.8) | 5, (13.2) | 30, (78.9) | 8, (21.1) |
|  | ***Moderate, (n=21)*** | 12, (57.1) | 9, (42.9) | 17, (81) | 4, (19) | 14, (66.7) | 7, (33.3) |
|  | ***Severe, (n=12)*** | 6, (50) | 6, (50) | 10, (83.3) | 2, (16.7) | 11, (91.7) | 1, (8.3) |
|  | ***p*** | n.s | | n.s | | n.s | |
| **Outpatient clinic visits** | ***Attending, (n=36)*** | 20, (55.6) | 16, (44.4) | 33, (91.7) | 3, (8.3) | 30, (83.3) | 6, (16.7) |
|  | ***Not attending, (n=45)*** | 22, (48.9) | 23, (51.1) | 34, (75.6) | 11, (24.4) | 32, (71.1) | 13, (28.9) |
|  | ***p*** | n.s | | n.s | | n.s | |
